# Energy and Environment: Challenges and Achievements in Rapid Urbanization

**DOI:** 10.1155/2013/594816

**Published:** 2013-09-18

**Authors:** Litao Wang, Hongxing Yang, Qunhui Wang, Shrestha Niranjan

**Affiliations:** ^1^Department of Environmental Engineering, School of City Construction, Hebei University of Engineering, Handan, Hebei 056038, China; ^2^Department of Building Services Engineering, The Hong Kong Polytechnic University, Hung Hom, Kowloon, Hong Kong; ^3^Department of Environmental Engineering, University of Science and Technology Beijing, Beijing 100083, China; ^4^Sagarmatha Engineering College, Kathmandu 44600, Nepal

Nowadays, more than half of the world's population lives in urban areas. It is forecasted that another 20% will move into cities within the next 40 years, most of which will occur in developing countries. It is predicted that the urbanization ratio will increase from the present 52.5% to 70% in 2020 in China. The rapid urbanization questions the sustainability. Cities require huge quantities of energy and materials which produce large quantities of waste products, raising the problems of energy shortage, water pollution, soil pollution, and air quality deterioration.

This special issue, of The Scientific World Journal focuses on those problems that we will face during this challenging process. The accepted topics are on the latest research of (1) energy efficient construction and renewable energy use in cities; (2) technologies in waste water treatment; (3) energy saving and pollution control of municipal solid waste incineration; and (4) urban air pollution chemistry, control, and relationship with energy use.

Nine papers selected from the submitted nineteen ones are published in this special issue. Some of the papers were unaccepted only because they were beyond the scope of this issue. We would like to express our gratitude to those authors for their support and contributions first in this opening. 

Out of the nine papers, two were review articles and others were research papers. The topic distributions are two for energy use and saving in cities, three for water pollution control and treatment, one for solid waste treatment, and three for air quality pollution and control. Below is a brief introduction to each of these papers in this special issue.

We will introduce the two important review papers at first. The paper “*Recent developments of electrochemical promotion of catalysis in the techniques of *DeNO_X_” by X. Tang et al. has summarized many valuable viewpoints related to electrochemical promotion of DeNO_X_. The removal of NO_X_ is of significant concern in reducing the pollution and protecting the environment at present in China. But up to now, the traditional ways of DeNO_X_ usually need a specific operating temperature window. Because of the situ controllability in the promoter concentration at the surface of a working metal catalyst, the EPOC provides an alternative way to improve catalysts performance. EPOC is quite a recent and remarkable part of catalysis, and hundreds of papers were written on this topic from the pioneering work of C.G. Vayenas in MIT and Patras University. This will be helpful for the development of new catalytic system with low operating temperature and high NO_X_ conversion for the removal of NO_X_. This paper is valuable for reducing the NO_X_ pollution of industrial exhaust gas and improving environmental quality.

The review paper “*The hydrolysis of carbonyl sulfide at low temperature: a review*” by S. Zhao et al. has systematically summarized the development of catalyst for catalytic hydrolysis of carbonyl sulfide (COS), reaction kinetics, and reaction mechanism and has analyzed some problems which need to be solved in the development of COS removal technology. COS, which widely exists in natural gas, petroleum gas, and water gas, is normally regarded as a significant poison for industrial catalysis. Furthermore, not only does COS lead to economic problems, but also affects the environment. It has been proven to be a major source of acid rain when oxidized to sulfur oxide and to promote photochemical reactions. Some methods have been developed for the removal of COS, including catalyzed hydrogenation, oxidation, and hydrolysis, in which the hydrolysis of COS is recognized as the most promising process due to mild reaction condition, cheapness, and higher conversion efficiency. This paper provides a possible guidance for the development of the organic sulfur removal technology as well as the air pollution control technologies.

In Z.-p. Zhang and F.-h. Du's paper “*Optimization and thermoeconomics research of a large reclaimed water source heat pump system,*” a large reclaimed water source heat pump is introduced as well as its design principles and techniques. Water source heat pump is considered among the most energy efficient technologies for providing heating and cooling in urban constructions. This paper has presented a detailed process of the design of a large, complicated system including a distributed heat pump heating system and a combination of centralized and decentralized systems. It provides an excellent example and reference for the future application of water source heat pump technology.

The paper “*Flow-field characteristics of high-temperature annular buoyant jets and their development laws influenced by ventilation system*” written by Y. Wang et al. has applied a numerical model to understand the flow characteristics of high-temperature annular buoyant jets and the development laws influenced by ventilation system to eliminate the pollutants effectively. Based on the detailed analysis using the appropriate method, the authors have drawn several remarkable conclusions that would be useful to the pollution control in industries.

C. Zhu et al.' paper “*Reduction of waste water in Erhai lake based on MIKE21 hydrodynamic and water quality model*” has applied the MIKE21 hydrodynamic and water quality model in Erhai Lake to simulate the water quality and water environment capacity. Erhai Lake is one of the most famous lakes in China and has suffered a lot from the water pollution in recent years. It may be of particular interest to the policy makers on water quality improvement of Erhai and other similar lakes in China.

The paper “*Research on phthalic acid esters removal and its health risk evaluation by combined process for secondary effluent of wastewater treatment plant*” by S. Li et al. has discussed the treatment efficiency of “coagulation-sedimentation-O_3_-biological sand filtration-GAC” combined process on phthalic acid esters in secondary effluent of municipal wastewater treatment plant, and the health risk is analyzed as well. It is informative and useful to the in depth treatment of municipal waste water in Chinese cities.

In W. Zhang et al.'s paper “*Characterization of urban runoff pollution between dissolved and particulate phases,*” the characteristics of urban runoff pollution between dissolved and particulate phases are discussed, using 12 rainfall events monitored in five typical urban catchments. It can provide useful information for the future control of the urban runoff pollutions in China.

 “*Formation of humic substances in weathered MSWI bottom ash*” is the only paper dealing with solid waste treatment in this special issue. It has presented an evaluation of humic substances content in the bottom ash of municipal solid waste incinerators and its affecting factors such as incubation time and temperature. It has concluded that the high temperature may be beneficial to the formation of humic acid, while low temperatures are conducive to the accumulation of fulvic acid. Two extraction reagents are compared as well, and the optimal formula is recommended.

The paper “*Quantifying the sources of the severe haze over the southern Hebei using the CMAQ model*” has applied the MM5-Models-3/CMAQ modeling system to quantify the source contributions to the PM_2.5_ concentrations in the southern Hebei cities, Shijiazhuang and Xingtai, which may be the top one and two polluted cities in China. This paper has examined the importance of each sector to the air pollution in these two cities. It may be of particular interest to the policy makers in the future air pollution control in this area.

